# Invasive ecosystem engineers threaten benthic nitrogen cycling by altering native infaunal and biofouling communities

**DOI:** 10.1038/s41598-020-58557-8

**Published:** 2020-01-31

**Authors:** L. W. Tait, A. M. Lohrer, M. Townsend, J. Atalah, O. Floerl, G. J. Inglis

**Affiliations:** 1 National Institute of Water and Atmospheric Research, 10 Kyle St, Riccarton, Christchurch, 8011 New Zealand; 2 National Institute of Water and Atmospheric Research, 10 Silverdale Road Hillcrest, Hillcrest, Hamilton, 3216 New Zealand; 3 Waikato Regional Council, 401 Grey St, Hamilton East, Hamilton, 3216 New Zealand; 4 Cawthron Institute 98 Halifax St E, The Wood, Nelson, 7010 New Zealand

**Keywords:** Biogeochemistry, Ecophysiology, Invasive species

## Abstract

Predicting the effects of invasive ecosystem engineering species in new bioregions has proved elusive. In part this is because separating biological effects from purely physical mechanisms has been little studied and yet could help predict potentially damaging bioinvasions. Here we tested the effects of a large bio-engineering fanworm *Sabella spallanzanii* (*Sabella*) versus worm-like structures (mimics) on gas and nutrient fluxes in a marine soft bottom sediment. Experimental plots of sediment in Hauraki Gulf (New Zealand) were used to test the hypothesis that ecosystem engineers negatively influence benthic ecosystem function through autogenic mechanisms, facilitating activity by biofouling organisms and competitive exclusion of native infauna. Enhanced physical structure associated with *Sabella* and mimics increased nitrogen fluxes, community metabolism and reduced denitrification from 23 μmol m^−2^ h^−1^ to zero at densities greater than 25 m^2^. *Sabella* plots on average had greater respiration (29%), NH_4_ release (33%), and greater NO_3_ release (52%) compared to mimics, suggesting allogenic (biological) mechanisms occur, but play a secondary role to autogenic (physical) mechanisms. The dominance of autogenic mechanisms indicates that bio-engineers are likely to cause significant impacts when established, regardless of fundamental differences in recipient regions or identity of the introduced bio-engineer. In the case of *Sabella spallanzanii*, compromised denitrification has the potential to tip the balance of net solute and gas exchanges and cause further ecological degradation in an already eutrophic system.

## Introduction

Ecosystem engineers create, destroy or otherwise modify habitats, often exerting a strong positive control on the structure and functioning of entire ecosystems^1,2^ and are recognised as some of the most damaging groups of invasive species^3,4^. However, when ecosystem engineers invade new environments the spectrum of physical, physico-chemical and ecological consequences makes it difficult to interpret the net balance of changes and quantify or predict impacts^4,5^. Even when much is known about a single introduced bio-engineering species, the effects on recipient ecosystems that vary across latitudes, regions, and local environmental gradients^6,7,8^ may, themselves, be highly variable^9,10^. Despite this context-dependence, impacts observed in one location are regularly used to infer potential impacts of introduced species at other locations^7,11^. In fact, serial invaders (e.g., *Styela clava*, *Sabella spallanzanii*, *Undaria pinnatifida*, *Sargassum muticum*, *Carcinus maenas, Caulerpa taxifolia*) are often used as model species for generalising traits that result in significant impacts to native ecosystems and are frequently included in ‘unwanted-organism lists’^12,13^. Elucidating the dominant mechanisms of ecological impacts from bio-engineers may demonstrate the utility of global lists of unwanted marine organisms for prioritising management efforts.

The impacts of introduced species on the ecology of recipient communities are most commonly reported as changes in native species populations, e.g., declines or extirpations of native species following outbreaks of non-native predators or competitors^3,9,14^. This approach is problematic when attempting to translate introduced species impacts on a global scale, as each recipient ecosystem has highly variable species assemblages and ecosystem structures. This is particularly true when native ecosystem engineers are affected by invasions. Research on native communities shows that the presence and abundance of ecosystem engineers (rather than species richness per se) is the predominant driver of ecosystem function (e.g., earthworms^1^, legumes^15^, marine bioturbators^16^). In many examples, ecosystem engineers that enhance the heterogeneity of above or below surface structures, contribute disproportionally to ecosystem functions, and may buffer anthropogenic stressors^17^. Introduced species that disrupt native ecosystem engineers will, therefore, have greater consequences on the functioning of native ecosystems^18^. In this sense, identifying the alteration of ecosystem functions performed by native functional groups (e.g., bioturbators, habitat-formers) may enable generalisation of introduced species impacts over broad scales, allow more accurate predictions of potential impact, and improve prioritisation of limited resources for management of marine bioinvasions.

One of the key ecosystem services performed by marine soft-sediment systems is the processing and potential loss of nitrogen^19^. The sediment-water interface is a focal point for critical ecosystem functions in benthic marine systems^16,20^, with dissolved nutrients (e.g., ammonium, phosphate) moving across the surface^21,22^ and particulate material accumulating beneath it. The balance of the fluxes in and out of the sediment is heavily modified by the seafloor biota which physically bioturbate the sediment matrix, altering the thickness and surface area of redox boundaries, and movement of water (i.e., active transport, bioirrigation^23^). The magnitude of exchange processes can be disrupted by elevated inputs of dissolved and particulate materials (e.g., through anthropogenic eutrophication or sedimentation^24^), or through changes to the biotic structure of benthic communities^23^. Species that are functionally unique (physically or physiologically) can cause major shifts in the balance of particulate and dissolved material with far-reaching consequences to the provision of ecosystem services^25^.

There are several examples of alteration of ecosystem functions by benthic invaders, for example: reduced nutrient uptake capacity by an introduced seagrass^26^; reductions in water column chlorophyll *a* by a introduced mollusc^27^; and reduced denitrification efficiency by a introduced tubeworm^28^. Yet the mechanisms responsible for shifts in ecosystem functioning are often less clear than the magnitude of change. Introduced species are capable of affecting ecosystem functioning through several mechanisms; direct trophic impacts^29^, indirect trophic impacts^30^, competition^31^ or facilitation of native or introduced organisms^32^, autogenic engineering and allogenic engineering^3^. Here we focus on the dominant mechanisms of ecosystem engineers: allogenic and autogenic. Autogenic engineering refers to the physical alteration of the environment by the bodies of ecosystem engineers (e.g., creation of biogenic habitat^33,34^). Allogenic engineering is the alteration of physical environmental properties via mechanical (e.g., filtering of particulate matter^35,36^ or chemical means (e.g., deposition of faeces^28^). For example, autogenic engineering can be realized through reductions or loss of key native macroinvertebrates through competitive exclusion^37^, which has the potential to modify the sediment-water interface and reduce the rate of several critical functions. Equally, introduced ecosystem engineers can facilitate both native and introduced species with potential for both positive^32^ and negative consequences^38^. Species that modify and create habitats provide a resource (e.g., space), which in the context of marine soft-sediment systems, has the potential to exclusively or simultaneously affect above sediment and below sediment organisms and processes. Autogenic engineering can also be realized through modification of the physical environment, which include changes to hydrodynamics that can influence boundary layer thickness, orbital bed velocities^39,40^, sediment accumulation/erosion^41^, and changes in light penetration to the benthos^35,36^. Allogenic engineering includes chemical modification effects such as increased production of organic rich biodeposits or enhanced excretion of ammonium^28^; interception and consumption of organic material^27^; and alteration of pore water solute concentrations and gradients (via oxygen respiration, ammonium excretion).

Experimental manipulations of introduced species and structural equivalents (i.e., mimics) allow the disentanglement of physical and biological effects^3^, while the simultaneous measurement of flux rates and community composition shifts may uncover the relative influence of chemical alteration by introduced species and loss of key processes when native species are displaced. Experiments separating the structural complexity of introduced species from metabolic processes have revealed that physical engineering is an important contributor to the impacts of introduced species^34,42^. However, to our knowledge no study has manipulated densities (i.e., a gradient of densities) of real and mimic structure-forming introduced species *in situ* and measured both flux rates and community composition shifts. There are, however, studies which have examined the relative contribution of living bivalves and non-living shells to denitrification rates, which indicate that internal tissues and shell material are colonised by denitrifying bacteria^43,44^. We postulate that an experimental design manipulating introduced species density and incorporating live introduced species and structural equivalents (mimics) will enable an assessment of the relative impact pathways of introduced bio-engineering species on nitrogen cycling of benthic systems, with broad relevance for inferring impacts to recipient ecosystems.

The tube-forming polychaete worm *Sabella spallanzanii* (Gmelin, 1791, Polychaeta: Sabellidae) was first detected in Waitemata Harbour, Auckland, New Zealand in 2009^45^ and has since spread rapidly across artificial, hard, and soft-sediment benthic habitats towards open coast environments. This species contrasts starkly to native communities in these areas which have been modified by fishing activities, sedimentation, and nutrient input, but also differ greatly from structure forming bivalve species that once occurred in high densities^46^. Current evidence suggests that *Sabella* greatly increases NH_4_ excretion and oxygen consumption, reduces denitrification efficiency, and is associated with shifts in infaunal composition^28^. These findings were interpreted as a response to the filtering capacity of *Sabella* and the biofouling supported by its tubes but separating the role of the living worm, biofouling attached to its tube and alterations to infauna is less straightforward. The rapid expansion observed in northern New Zealand has important implications for the greater Hauraki Gulf marine ecosystem surrounding Auckland (New Zealand) which has seen dramatic increases in nutrient additions from land-use change^47^. Threats to nutrient removal capacity at the sediment-water interface may exacerbate eutrophication and hypoxia issues in the region^47^. Disentangling the relative mechanisms of impact, including critical density thresholds, has important implications for managing the effects of organic nutrients on coastal ecosystems, particularly the setting of acceptable limits of nitrogen inputs from anthropogenic activities. Furthermore, identifying the dominant ecosystem engineering mechanisms will help determine the potential for broader inference of introduced species impacts on global scales, where impacts related to reductions in native engineers, or facilitation of native or introduced species have context specific impacts, but impacts related to physical and chemical alteration have general relevance to benthic ecosystems.

**Figure 1:**
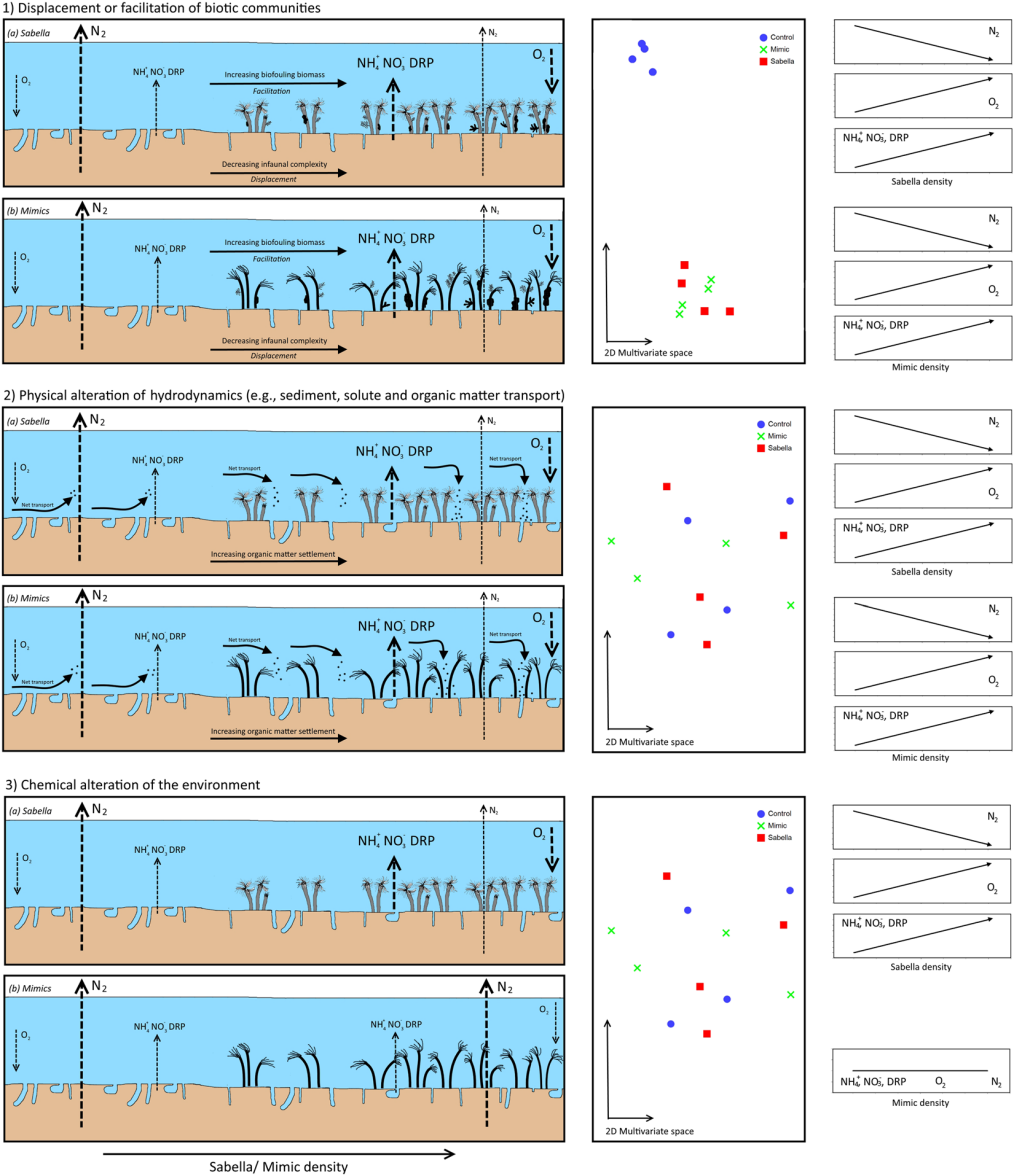
Model impact mechanisms of *Sabella spallanzanii* (**a**) and artificial mimics (**b**) on flux rates and community composition across density gradients; (1) Displacement or facilitation of biotic communities by presence of above sediment structure formers; (2) Physical alteration of hydrodynamic transport of sediments, solutes or organic matter by *Sabella* and mimics with no changes in community composition and both *Sabella* and mimics impact flux rates, but with unknown consequences; (3) Chemical alteration by *Sabella* has no impact on community composition but affects flux rates in *Sabella* plots only.

The three theoretical models of impact associated with introduced ecosystem engineers relate to two mechanisms of autogenic engineering (‘1’ Displacement or facilitation of biotic communities; ‘2’ Alteration of the physical environment) and one mechanism of allogenic engineering (‘3’ Chemical alteration). Each mechanism is expected to produce a unique combination of responses across live bio-engineers (Fig. 1a for models ‘1’, ‘2’, and ‘3’) and mimics (Fig. 1b for models ‘1’, ‘2’, and ‘3’) and would differentially impact flux rates and community composition if acting alone. For impact mechanism ‘1’, displacement or facilitation of biotic communities, it would be expected that the presence of live *Sabella* (Fig. 1, model ‘1’ a) and mimics (Fig. 1, model ‘1’ b) would affect community composition and subsequently flux rates. For impact mechanism ‘2’, alteration of hydrodynamic regimes, both *Sabella* (Fig. 1, model ‘2’ a) and mimics (Fig. 1, model ‘2’ b) would each impact flux rates through elevated accumulation of organic material^39,40^, but would have minimal consequences on biotic community composition. Impact mechanism ‘3’, chemical alteration, would affect flux rates in the live *Sabella* treatment only (Fig. 1, model ‘3’ a) and not mimics (Fig. 1, model ‘3’ b), but with no differences in community composition between treatments. Here we use an experimental density gradient of *Sabella* and mimics and examine shifts in flux rates and key fauna to test these three theoretical models of impact mechanisms. We hypothesize that the autogenic physical impact pathways will be the dominant mechanism of impact (model ‘1’ and ‘2’).

## Results

**Figure 2:**
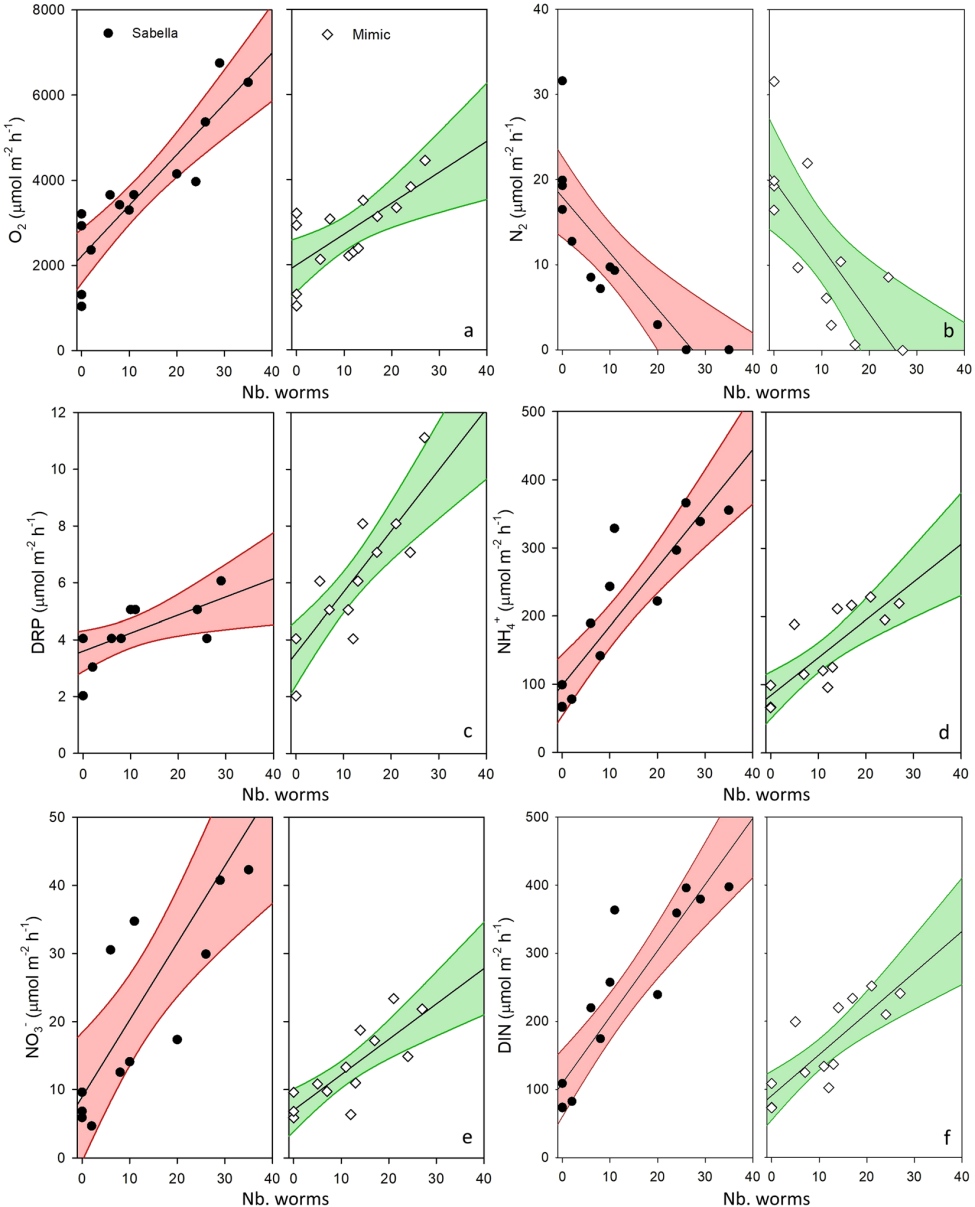
Response of biogeochemical fluxes to *Sabella* and mimics across a gradient of densities (Nb. worms per chamber). Responses fitted by linear regressions including 95% confidence intervals Community respiration, i.e., oxygen flux (**a**), denitrification rates, i.e., N_2_ flux (**b**), dissolved reactive phosphorus flux (**c**), ammonium flux (**d**), nitrate flux (**e**), and combined dissolved inorganic nitrogen flux (**f**) shown separately for ‘*Sabella*’ and ‘Mimic’ treatments across density gradients.

Nutrient cycling and community oxygen consumption rates were generally greater in the presence of live *Sabella spallanzanii* (‘*Sabella*’) compared to structural equivalents (‘Mimics’). Flux rates of oxygen, nitrogen, and phosphorus had a linear positive relationship with worm density, but denitrification rates declined with increasing densities of *Sabella* and mimics (Fig. 2). There were, however, some key differences in solute fluxes between *Sabella* and mimics. Community respiration (O_2_, Fig. 2a; Table 1) and NO_3_ flux rates were greater in *Sabella* plots (Fig. 2e; Table 1), while dissolved reactive phosphorus (DRP) flux rates were greater in plots with mimics (Fig. 2c). *Sabella* density had little effect on DRP but increased with density of mimics (Fig. 2c, Table 1). Ammonium (NH_4_, Fig. 2d, Table 1) and total DIN flux (Fig. 2f, Table 1) increased with density of both *Sabella* and mimics (Fig. 2b, Table 1), but the significant density × treatment interaction suggested that the increase in fluxes of *Sabella* plots was greater than mimics. Rates of denitrification declined with increasing density of *Sabella* and mimics and at densities greater than 20–25 worms per m^2^ denitrification rates approached zero for both *Sabella* and mimics (Fig. 2b).

**Table 1:** Linear model results for fluxes of O_2_, N_2_, DRP, NH_4_ and NO_3_ and DIN. Model coefficients included densities of *Sabella* (or mimics), experimental treatment (*Sabella* or mimics) and their interaction, chlorophyll *a* concentration (Chla), phaeopigment concentration (Phaeo), sediment organic content (Organics), proportion of two sediment grainsize fractions (Gravel and Clay). Results from model selection presented, and statistically significant results presented in bold and italics.

Model coefficients	O_2_		N_2_		DRP		NH_4_^+^		NO_3_^−^		DIN	
	t	p	t	p	t	p	t	p	t	p	t	p
Intercept	−***6.2***	<***0.001***	0.4	0.7	***6.0***	<***0.001***	***3.5***	***0.002***	2.0	0.06	***3.5***	***0.002***
Density	−***4.3***	<***0.001***	***−6.5***	<***0.001***	***6.2***	<***0.001***	***3.9***	<***0.001***	***2.1***	***0.049***	***3.9***	<***0.001***
Treatment	−0.5	0.6	—	—	0.4	0.7	0.8	0.5	0.4	0.7	0.8	0.4
Density × Treatment	***−3.1***	***0.005***	—	—	−***4.3***	<***0.001***	***2.1***	***0.047***	***2.1***	***0.048***	***2.3***	***0.03***
Chla	—	—	1.9	0.07	—	—	—	—	—	—	—	—
Phaeo	—	—	—	—	−***2.4***	***0.03***	−1.4	0.2	—	—	−1.5	0.2
Organics	***3.7***	***0.001***	—	—	—	—	—	—	—	—	—	—
Gravel	—	—	—	—	−1.9	0.07	—	—	—	—	—	—
Clay	—	—	***2.5***	***0.02***	—	—	—	—	—	—	—	—
**Combined model**												
r^2^		0.85		0.73		0.76		0.81		0.68		0.83
Adjusted r^2^		0.83		0.69		0.7		0.79		0.64		0.8
F_5,22_		33.3		18.9		13.9		25.9		16.8		28
p		<0.001		<0.001		<0.001		<0.001		<0.001		<0.001

Multiple linear regression results revealed that densities of *Sabella* and mimics, or interactions between treatment and density, were the primary drivers of changes to ecosystem functions (Table 1). Model selections revealed variation between flux variables, with O_2_ flux explained by density, and a significant treatment × density interaction. Sediment organic content also explained O_2_ flux, or co-varied with experimental treatments. N_2_ flux was explained by density, with the clay composition also explaining or co-varying with treatments. DRP flux was driven by density, and a treatment × density interaction, with phaeopigments also explaining or co-varying. NH_4_ flux was explained by density and a treatment × density interaction. Like NH_4_, NO_3_ was explained by density, and a significant treatment × density interaction. Total DIN showed similar explanatory variables as NH_4_, likely driven by the much higher flux rates of NH_4_ compared to NO_3_. Although there was no significant relationship between chlorophyll *a* and density, there was a significant treatment affect, with *Sabella* plots exhibiting elevated sediment chlorophyll *a* (Supplementary Fig. S1).

**Figure 3:**
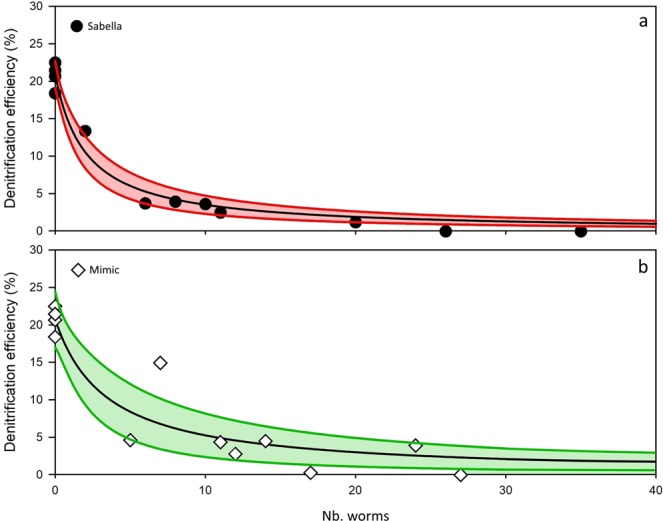
Denitrification efficiency across densities of *Sabella* (**a**)and mimics (**b**). Data and 95% confidence intervals fitted by hyperbolic decay function.

Unlike other flux variables, denitrification efficiency (the proportion of nitrogen fluxed into the water column as N_2_ compared with the total dissolved inorganic nitrogen [Ross *et al*.^28^]) was not affected linearly with increasing worm density and showed a fast decline with increases in *Sabella* and mimic densities (Fig. 3). Beyond approximately 10 worms per m^2^, denitrification efficiency fell below 5% for *Sabella* plots. Mimics had a broader range of denitrification efficiency with increasing density, but beyond densities of 20 per m^2^ denitrification efficiency was below 5%.

**Figure 4:**
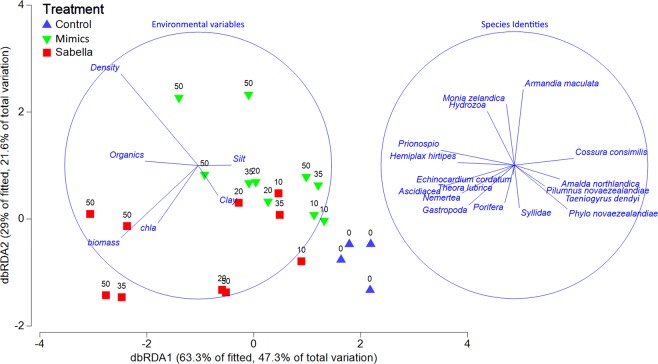
Distance based linear models (DistLM) of flux rates as response variables and the influence of environmental parameters and epibiota as explanatory variables. Flux rate data was normalised and dissimilarity matrix calculated using the Euclidean distance metric. Environmental variables were selected using the backwards selection procedure, and AIC selection criteria.

Combined flux profiles (i.e., the multivariate response of combined fluxes; NH_4_, NO_3_, N_2_, O_2_, DRP) showed high separation between treatments, with much greater separation between mimics and *Sabella*. The integrated flux profiles of several plots with high densities of living *Sabella* (establishment densities of 35–50 worms per m^2^) were noticeably separated from all other plots. Overall there was a significant difference in the flux profiles between treatments (Psuedo-F_1,23_ = 13.5, p(perm) = 0.001), and density (Psuedo-F_1,23_ = 2.6, p(perm) = 0.03). Flux rates of *Sabella* plots were explained by higher biomass, phaeopigments, chlorophyll *a* and smaller sediment fractions (Fig. 4, Supplementary Table S2). Variation in flux rates was also related to variations in species assemblages (Fig. 4). Flux rates of control plots were significantly associated with the presence of a burrowing crab (*Pilumnus novaezealandiae*), a burrowing holothurian (*Taeniogyrus dendyi*), and the brushworm *Phylo novaezealandiae* (Fig. 4). Conversely flux rates of plots containing *Sabella* and mimics were associated with biofouling organisms such as ascidians, hydroids and porifera (Fig. 4). Flux rates of mimic plots were also associated with the small polychaete family Myriochele and the burrowing heart urchin *Echinocardium cordatum* (Fig. 4).

Despite shifts in species identity across treatments, there were neutral trends in species richness and biodiversity metrics (Shannon diversity and Pielou’s evenness) with increasing densities (Supplementary Fig. S3). There was, however, a significant increase in overall biomass with increasing densities of *Sabella*, and mimics (Supplementary Fig. S3, Panel A). Although the accumulated biomass with increasing densities of *Sabella* included the live worms themselves, the strong trend of increasing biomass with density of mimics showed the large contribution of biofouling organisms (e.g., ascidians, sponges and hydroids attached to *Sabella* or mimics).

## Discussion

Six months after establishing experimental density gradients of non-native fan worms (*Sabella spallanzanii*) and structural analogues (mimics), several changes to seafloor sediment characteristics, bioturbating species and ecosystem functions were observed. Notably, effluxes of dissolved inorganic nitrogen (ammonium and nitrate) across the sediment-water interface, and rates of community respiration increased significantly with worm density. In contrast, rates of denitrification (a microbially mediated ecosystem function that eliminates bioavailable inorganic nitrogen from the system) declined. This was the first study of the effects of *Sabella* on ecosystem functioning in New Zealand, where it was first recorded 10 years prior. Our findings were consistent with effects predicted for invasive ecosystem engineers^4^ and similar to results of experiments on *Sabella* conducted in Port Phillip Bay, Australia by Ross *et al*.^28^. We observed much greater reductions in denitrification, and reduced flux of dissolved reactive phosphorus compared to Ross *et al*.^28^. However, much like the findings from Guy-Haim *et al*.^4^ there were neutral effects of an invasive ecosystem engineer on several biodiversity metrics, likely associated with simultaneous reductions in infaunal biodiversity and gains in biofouling biodiversity.

Invasive species with ecosystem engineering traits have been linked to high magnitude ecological impacts^3,14,42^. In our study, *Sabella* and structural equivalents (mimics) altered the composition of benthic infauna and epibiota and facilitated tube fouling organisms, which was strongly associated with changes to biogeochemical cycling. Our findings support the autogenic engineering hypotheses that changes in habitat availability for other organisms is a dominant component of structure forming introduced species impacts. There was evidence of reductions in large bioturbating species by *Sabella* and mimics and increasing biofouling biomass (impact mechanism ‘1’). Interestingly in this example the facilitation of largely native fouling organisms (exceptions were two introduced ascidians *Symplegma brackenheilmi* and *Styela clava*) was associated with negative consequences to important ecological functions (e.g., denitrification). However, the process of denitrification is driven by bacterial activity and it is unclear from this study how bacterial denitrification is being impacted by *Sabella* or mimics.

Rates of denitrification decreased linearly with increasing structures, both living (*Sabella*) and analogues (mimics), while denitrification efficiency declined exponentially. This contrasts with the positive linear increase in sediment denitrification with increasing bivalve biomass (clams, *Ruditapes philippinarum*) and no net change to denitrification efficiency^48^. Likewise, denitrification potential was either unchanged or increased in the presence of the burrowing bivalve *Austrovenus stutchburyi*^49^. Both of these examples include burrowing clams which have little above sediment structure, however, another bivalve *Atrina zelandica* which protrudes from sediments can also potentially contribute to enhanced denitrification^50,51^. Density gradients of above-sediment, structure-forming bivalves *Atrina zelandica* have also been shown to physically influence sediment dynamics through reductions in resuspension^39^, yet similar mechanisms of physical changes between bivalve molluscs and tube-forming polychaetes (i.e., *Sabella*) are having contrasting biogeochemical outcomes.

Disentangling cause and effect when examining changes in fluxes and biogeochemistry is difficult due to the complexity of linked interactions and opposing processes (e.g., photosynthesis and respiration). The addition of live *Sabella* likely affected solute fluxes in both direct (respiration, excretion) and indirect ways (altered hydrodynamics, sediment characteristics, and macro- and micro-organism communities), but the similarly large changes in benthic fluxes associated with mimics suggest that autogenic engineering mechanisms dominate. Species with characteristics of ecosystem engineers modify the physical environment through two mechanisms, autogenic and allogenic engineering^1^. Autogenic engineering is the alteration of physical aspects of the environment by the bodies of the engineers themselves (e.g., alteration of flow regimes by *Sabella* tubes or provision of habitat for other organisms), whereas allogenic engineering is the alteration of physical environmental properties via mechanical or chemical means (e.g., extraction of particulate material by *Sabella*)^1^. Shifts in bioturbating ecosystem engineers (e.g., *Echinocardium cordatum*^16^) suggest the autogenic influence of the introduced species (*Sabella*) may be partially mediated through shifts in the composition of native fauna, flora and microbiota^52^ which play important functional roles in nutrient cycling, particularly removal of bioavailable nitrogen (i.e., denitrification). Alterations of infaunal community composition was observed by Ross *et al*.^28^, and our results suggest this may be associated with a combination of crowding and interception of suspended particulate organic material by *Sabella* and biofouling on its tubes. The dominance of autogenic impact mechanisms may have broader implications for understanding the generality of impacts from bioinvasions and suggests that bio-engineering species which impact one region are highly likely to affect other bioregions.

Multivariate analysis of combined flux profiles (i.e., NO_3_, NH_4_, DRP, O_2_ and N_2_) showed that plots of living *Sabella* separated from plots of mimics indicating that the sum of solute changes was uniquely affected by *Sabella*. It is possible, that nuances of packaging and deposition of psuedofaeces^53^, or suitability of the tube or soft tissue for colonisation by nitrifying or denitrifying bacteria^43,44^ may be responsible for the separation of flux profiles between live *Sabella* and mimics. There is also evidence that *Sabella* produce a mucus with antibacterial properties, which could potentially upset the balance of nitrifying/denitrifying bacteria^53^, yet our study suggests that if this mechanism operates, it is secondary to the physical influence of tube structure. Combined, our results reveal that structure-forming introduced species represent a major threat to ecosystem functioning of marine ecosystems, as has been postulated by others^3,4,14^. Such traits are likely to cause significant impacts in many recipient ecosystems.

Nutrient remediation through denitrification is a critical service provided by marine benthic communities^54^. It has been proposed that *Sabella* could be used as a bioremediator of waste inputs from aquaculture and sewage due to its ability to remove organic matter and bacteria from the water column^55,56^. However, our study (and also Ross *et al*.^28^) urge extreme caution in this respect, as *Sabella* can compromise nitrogen cycling of sediments, and may in fact have a net negative effect on nutrient remediation. While denitrification is performed by denitrifying bacteria, the magnitude of N_2_ release is mediated by key infaunal biota^16,57–59^. Disruption of these key infauna by invasive habitat-structuring species has the potential to exacerbate problems associated with anthropogenic nutrient additions but has received almost no attention compared to the influence of terrestrial sediment and nutrient inputs^24,60–62^. New Zealand’s nitrogen balance worsened more than any other OECD country between 1998 and 2009, principally due to farming intensification^63^. Spread of *Sabella* throughout the Hauraki Gulf may result in greater nitrogen retention and thus potentially runaway eutrophication and increasing likelihood of hypoxic events. Compromised denitrification, increased inorganic matter content, and enhanced oxygen consumption have the potential to tip the balance of net solute and gas exchanges and cause further ecological degradation in an already eutrophic system^47^. To prevent further harm, resource managers may ultimately have to reduce inputs of nitrogen e.g., set lower limits of nutrient discharge, thus impacting terrestrial productivity with associated economic and social impacts.

Our results indicate a dramatic decline in denitrification efficiency at relatively low densities of *Sabella* and mimics, but other flux parameters had a linear response to density. While some solute flux rates (NO_3_, NH_4_) were affected by relatively low densities, N_2_ release, DRP release, and O_2_ consumption were greatly affected by worm densities above 10 per m^2^. The loss of these key ecosystem functions and subsequent consequences to wider ecosystem health provides significant incentive for management efforts to minimise the density of *Sabella*, and therefore, promote ecosystem functioning of benthic communities. There are, however, few feasible management approaches which don’t equally damage native epibiota, and the high levels of arsenic in *Sabella spallanzanii* tubes^64^ may deter native predators leaving them to spread unchecked. Given the potential cost of ongoing management over long periods, there is even greater incentive for sustained eradication efforts of bio-engineering introduced species at the early stages of incursion. *Sabella* has enormous potential for economic impacts to the aquaculture industry^65^, and our study suggests that the ecological impacts may be equally severe.

## Methods

### Study site

Field work for this research was performed by SCUBA divers in a subtidal (9 m depth) soft-sediment seafloor habitat in Rangitoto Channel (174.83877, 36.81350; North Island, New Zealand), approximately 10 km from where the first North Island populations of *Sabella* were recorded in 2009^45^. The study site is protected from open ocean swell but has strong tidal currents (up to 0.9 m/s) and is periodically subjected to wind-driven waves.

### Experimental design

To understand the effects of *Sabella* on soft-sediment ecosystem functions at the study site, live *Sabella* were collected from the Rangitoto Channel seafloor and transplanted into experimental plots at specific densities. To distinguish between the biological effects of live *Sabella* and the physical effects of the structure created by their tubes, structural ‘mimic’ *Sabella* were also transplanted into plots at the site. The *Sabella* mimics, made of flexible bungee cord, were similar to real *Sabella* in terms of length (25–30 cm), thickness (8 mm dia.), appearance (complete with faux feeding tentacles at the anterior end), clustering (singles, pairs, larger groupings), and movement characteristics (e.g., bending and swaying with water motion). Bungee cord was not tested for toxicity but was selected for inert materials (natural rubber and cotton). Steel pegs were used to anchor mimics and live worms in place when necessary. For the live *Sabella* treatments, only healthy undamaged worms with intact tubes were transplanted. Many of the *Sabella* collected at the study site were attached to mollusc shell fragments (sometimes several *Sabella* individuals per shell), and thus treatments were created by arranging naturally occurring clusters of worms into experimental plots to achieve the target densities.

In total, twenty-four 1 m^2^ experimental plots were established at the study site in September 2017. Plots were positioned two meters apart from one another along two perpendicular 30 m transects with a common vertex (n = 12 plots per transect). Live worms and *Sabella* mimics were transplanted to the plots at densities of 0, 10, 20, 35, and 50 per m^2^. Treatment positions were randomly interspersed along the transects. There were no mixed treatments containing both live and mimic *Sabella*. Experimental gradients in the densities of live and mimic *Sabella* were created by establishing four control plots (i.e., steel pegs only, with no live or mimic *Sabella*); two plots each with 10, 20 and 35 live *Sabella*; two plots each with 10, 20 and 35 mimic *Sabella*; and four plots each with 50 live and 50 mimic *Sabella*. Extra replicates in the end member treatments (i.e., four replicates each in the “0” and “50” treatments, rather than two each) limited the influence of outlying data points in these treatments and therefore provided greater statistical power. Given the potential for density-dependent mortality over time in the live *Sabella* treatments, the design also increased the likelihood of an even distribution of densities of live worms across the gradient at the end of the experiment when ecosystem functions and various environmental metrics were assessed.

### End of experiment sampling

In March 2018, approximately six months after establishing experimental treatments, plots at the study site were re-sampled. The aim was to quantify the effects of *Sabella* on benthic rates and processes (assessed by examining fluxes of dissolved solutes across the sediment-water interface). and on sediment and benthic community characteristics that are known to interactively influence those rates and processes.

Fluxes of dissolved oxygen, elemental nitrogen (N_2_) and inorganic N and P were quantified using benthic incubation chambers, as described by Lohrer *et al*.^16,66^. One square aluminium chamber base (50 cm × 50 cm, wall 15 cm high) was positioned in the centre of each experimental plot. Chamber bases were pressed ~5–7 cm deep into the sediment, enclosing a 0.25 m^2^ patch of sediment and a known number of live or mimic *Sabella*. A DOpto dissolved oxygen logger sampling at 1 minute intervals and a SeaBird Electonics water pump (pulsed, non-directional stirring) was positioned on the interior edge of each base.

The next morning, watertight Perpex lids were clamped onto each chamber base to initiate incubations, sealing ~30 L of bottom seawater in with enclosed sediments in each plot. Opaque shade clothes were used to cover the chambers (i.e., dark incubations in all plots). Although DO was measured at 1-minute intervals with the loggers, two 60 ml water samples were also collected manually from each chamber (through syringe-activated sampling ports) to track changes in DO and other dissolved solutes (elemental nitrogen, ammonium, nitrate + nitrite, and phosphate) during the 3 hour incubation period.

Immediately upon surfacing, the concentration of DO in one of each pair of water samples was assessed using a handheld dissolved oxygen meter (YSI ProODO). The sample was then filtered across 0.8 mm Whatman glass fibre filter and stored frozen until later analysis of dissolved inorganic N and P (Astoria-Pacific 300 series segmented flow auto-analyser with detection limits of 1 mg/m3 for N and P). The second sample was decanted into triplicate 15 ml glass ‘exetainers’, which were filled to overflowing, preserved with 1 drop of mercuric chloride (HgCl_2_), and capped without any air bubbles or headspace. The capped exetainers were stored in a water bath (at 1–4 C below the temperature of ambient bottom water at the time of sample collection) until analysis for N_2_ gas concentration and [N_2_]/[Ar] ratios using membrane inlet mass spectrometry (MIMS^67^).

In all cases, fluxes of solutes were calculated as concentration change during incubation (µmol/L) times chamber volume (30 L), divided by elapsed incubation time (h) and area of sediment enclosed (0.25 m^2^).

At the end of the chamber incubations and after the lids had been lifted, two small sediment cores (3 cm internal diameter, 2 cm deep) were collected from each experimental plot: one for sediment grain size and organic matter content analysis^68,69^, the other for sediment chlorophyll *a* and phaeopigment content^70^. Two larger cores (12 cm internal diameter, 10 cm deep) were collected from each plot to sample benthic macrofauna communities. Macrofaunal samples were sieved across a 0.5 mm mesh screen; preserved in 70% isopropyl alcohol, stained with Rose Bengal, sorted, and identified to the lowest practicable level. All fauna >5 mm remaining in the chamber bases, including live *Sabella* and the *Sabella* mimics, were collected in mesh bags and brought to the surface for enumeration. Biomass (blotted wet weight) of the transplanted *S. spallanzanii* present in each chamber, and biomass of the organisms attached to the live and mimic *Sabella*, was also quantified.

### Statistical analysis

Various univariate and multivariate statistical procedures were used to assess the influence of *Sabella*, six months after establishing experimental gradients in the densities of live worms and mimics. The effects of manipulated variables and environmental co-variables (grain size, OM content, sediment pigments, macrofauna) on fluxes of dissolved solutes, oxygen, ammonium, nitrate, total DIN (dissolved inorganic nitrogen), phosphate and N_2_ were analysed. Although gradients in the densities of live and mimic *Sabella* were categorical at the 1 m^2^ plot scale at the time of experimental setup (i.e., 0, 10, 20, 35 and 50 worms/mimics per plot in September 2017), the 0.25 m^2^ chambers (n = 24) did not enclose all of the worms/mimics transplanted into a plot, and mortalities were expected to occur in some of the live *Sabella* treatments. Therefore, densities of worms/mimics present in the chambers at the time of sampling (March 2018) were used as continuous predictor variables in analyses of flux variables. Multiple linear regression models were constructed in RStudio^71^ to partition the sources of variation in the solute flux response variables. Predictor variables included the experimental treatment (*Sabella* vs. mimics), density of worms (continuous predictor), sediment chlorophyll *a* content, sediment phaeopigment content, sediment organic content, and the contribution the large and small grainsize fractions to sediment composition (gravel and clay). Model predictor selection was performed by stepwise AIC selection criteria (forwards and backwards). Model assumptions were checked using fitted vs residual, QQ, and scale location plots. Diagnostics showed linear relationships (fitted vs residuals), residuals were normally distributed except for a few outliers (QQ), and data showed homoscedasticity (scale-location plots).

Alongside flux rates, the denitrification efficiency was also calculated to assess the relationship between DIN diverted away from benthic process through direct ammonia excretion (Ross *et al*.^28^). DE (%) was defined as the proportion of N fluxed into the water column as N_2_ compared with the total dissolved inorganic N (DIN; NH_4_, and NO_3_) released into the water column and was calculated from the following:

$$DE( \% )=\frac{{N}_{2}}{{N}_{2}+DIN}\cdot 100$$

where N_2_ is the number of moles of N fluxed into the water column as N_2_, and DIN is the number of moles of DIN fluxed into the water column (Ross *et al*.^28^).

Distance based linear models (DistLM) were used to examine the relative influence of several explanatory environmental variables and community composition on flux rates. Flux rates were normalised and resemblance matrices were calculated using Euclidean distance similarities to produce a multivariate metric of flux profiles (i.e., the combined flux response of each replicate plot). The explanatory variables were; above sediment biomass; sediment chlorophyll *a* content; sediment phaeopigment content; sediment organics content; and proportion of seven grainsize fractions in the sediment. Distance based linear models were performed using the backward selection method and the AIC selection criteria. Flux rates were plotted using distance-based redundancy analysis (db-RDA), with the species and environmental variables driving variation plotted separately as vector overlays. The influence of the experimental factors, density and treatment on flux profiles were analysed using permutational analysis of variance (PERMANOVA). Both treatment (2 factors ‘Sabella’, and ‘Mimic’), and density (5 factors; 0, 10, 20, 35, and 50) were treated as fixed factors. “Analysis was performed on a Euclidean distance” resemblance matrix and used a reduced model with 999 permutations. All multivariate analyses were performed using the statistical package PRIMER 7.

The datasets generated during and/or analysed during the current study are available from the corresponding author on reasonable request.

## Supplementary information

Supplemental information.
