# Supplementary material for: Invasive ecosystem engineers threaten benthic nitrogen cycling by altering native infaunal and biofouling communities
Source: Sci Rep. 2020 Jan 31;10:1581. doi: 10.1038/s41598-020-58557-8 (PMC6994685; doi:10.1038/s41598-020-58557-8)
Supplement: Supplementary file 1 — Supplemental information. [file 41598_2020_58557_MOESM1_ESM.pdf]

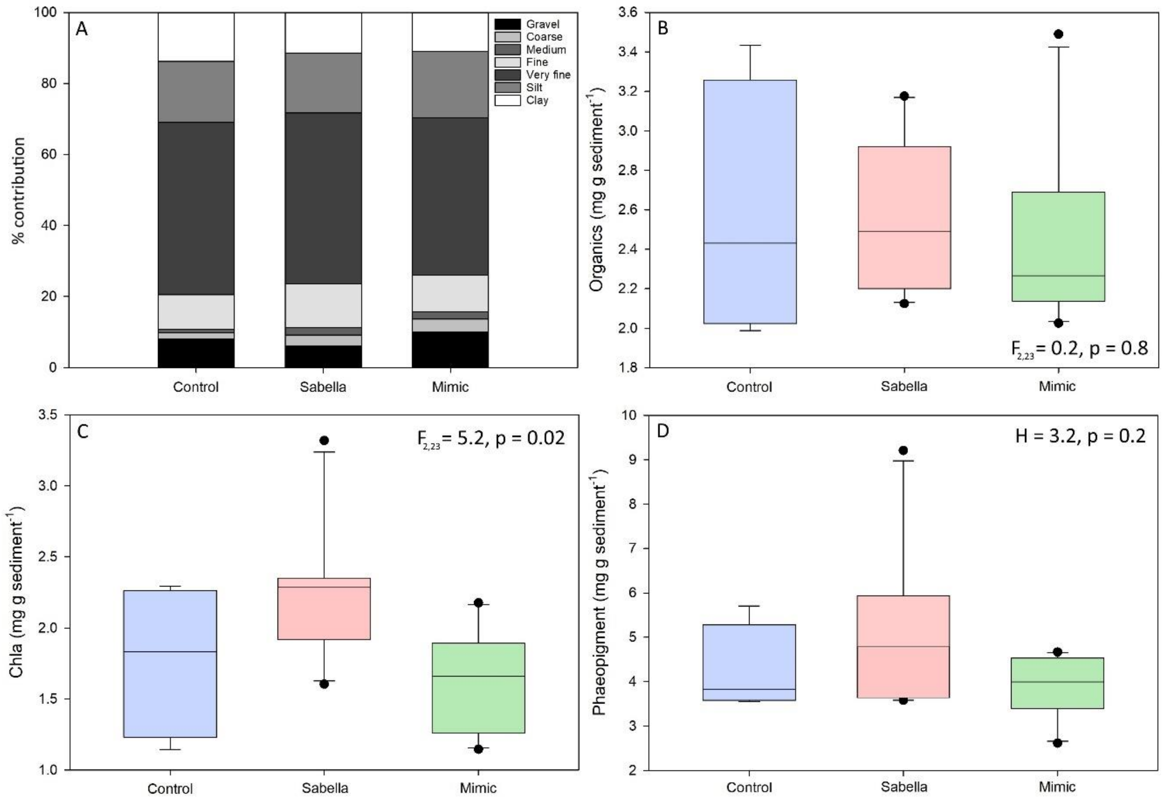

Supplementary Fig. S1. Influence of experimental treatments ('Control', 'Sabella' and 'Mimic') on sediment grainsize composition (A), sediment organic content (B), sediment chlorophyll a content (C), and sediment phaeopigment content (D). One-way ANOVA results (or Krsukal-Wallis ANOVA on ranks) comparing treatment are shown on graphs B, C and D.

Supplementary Table S2. Marginal tests of environmental parameters to variation in the combined matrix of flux responses. Marginal tests performed using the AIC selection criteria in a backwards procedure.

Variable	SS(trace)	Pseudo-F	P	Proportion
Biomass	46	14.7	0.001	0.4
Density (Sabella or mimics)	45.4	14.4	0.001	0.4
Chlorophyll a	18.5	4.2	0.008	0.2
Phaeopigments	10.6	2.2	0.09	0.09
Organics	25.2	6.2	0.003	0.2
Gravel	6.8	1.4	0.3	0.06
Coarse	8.6	1.8	0.15	0.08
Medium	6.7	1.4	0.3	0.06
Fine	2.7	0.5	0.6	0.02
Very-fine	8.2	1.7	0.2	0.07
Silt	2.5	0.5	0.7	0.02
Clay	5.1	1.0	0.4	0.04

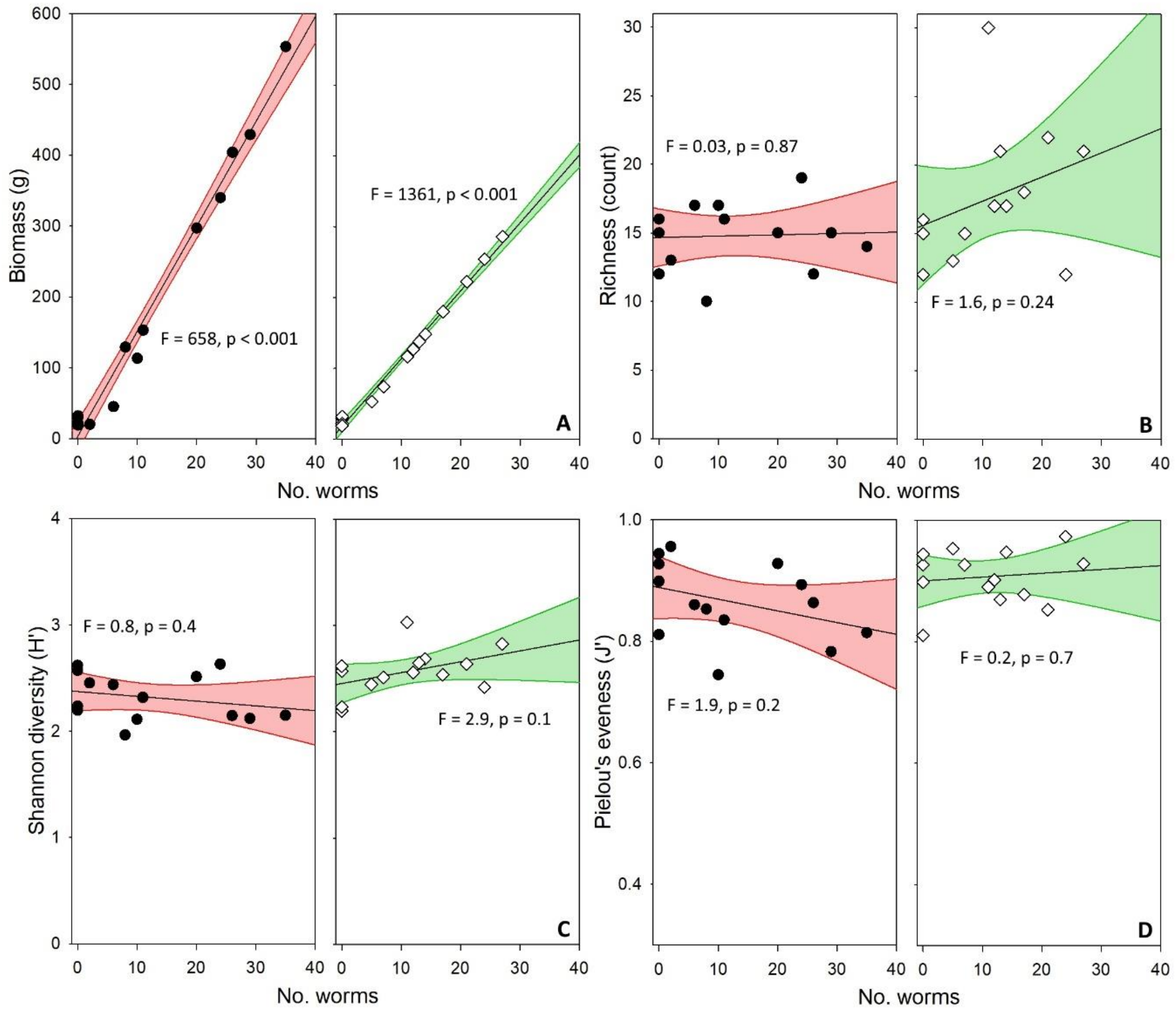

Supplementary Fig. S3. Response of biomass (A) and biodiversity metrics (richness, B; diversity, C; and evenness D) to real and mimic worms across a gradient of densities (Nb. worms per chamber). Responses fitted by linear regressions including 95% confidence intervals. Analysis of linear regressions shown for each panel.
